# Emerging Therapeutic Targets and Future Directions in Advanced Gastric Cancer: A Comprehensive Review

**DOI:** 10.3390/cancers16152692

**Published:** 2024-07-29

**Authors:** Margherita Ratti, Elena Orlandi, Ilaria Toscani, Stefano Vecchia, Elisa Anselmi, Jens Claus Hahne, Michele Ghidini, Chiara Citterio

**Affiliations:** 1 Oncology and Hematology Department, Piacenza General Hospital, Via Taverna 49, 29121 Piacenza, Italy; e.orlandi@ausl.pc.it (E.O.); i.toscani@ausl.pc.it (I.T.); s.vecchia@ausl.pc.it (S.V.); e.anselmi@ausl.pc.it (E.A.); c.citterio@ausl.pc.it (C.C.); 2 Division of Molecular Pathology, The Institute of Cancer Research, Sutton, London SM2 5NG, UK; hahnejen@yahoo.de; 3 Medical Oncology Unit, Fondazione IRCCS Ca’ Granda Ospedale Maggiore Policlinico, 20122 Milan, Italy; michele.ghidini@policlinico.mi.it

**Keywords:** metastatic gastric cancer, molecular targets, immunological targets, ctDNA, CAR-T cells, cancer vaccines

## Abstract

**Simple Summary:**

Metastatic gastric cancer (GC) remains a major clinical challenge due to scarce treatment options and an ominous prognosis, often diagnosed at later stages. Recent progress in identifying molecular targets and integrating immune checkpoint inhibitors with chemotherapy has transformed treatment strategies. The new classification of GC, based on immunologic and molecular markers, has drastically changed the therapeutic approach, focusing on personalized target-based treatments. This review summarizes molecular and immunological targets currently available and explores future approaches like circulating tumor DNA (ctDNA) monitoring, targeting fibroblast growth factor receptor (FGFR) and MET pathways, chimeric antigen receptor (CAR)-T cells, and cancer vaccines for their potential to improve understanding and treatment of this disease.

**Abstract:**

Metastatic gastric cancer (GC) still represents a critical clinical challenge, with limited treatment options and a poor prognosis. Most patients are diagnosed at advanced stages, limiting the chances of surgery and cure. The identification of molecular targets and the possibility of combining immune checkpoint inhibitors with chemotherapy have recently reshaped the therapeutic landscape of metastatic gastric cancer. The new classification of gastric cancer, mainly based on immunologic and molecular criteria such as programmed cell death 1 (PD-1), microsatellite instability (MSI), and human epidermal growth factor receptor 2 (HER2), has made it possible to identify and differentiate patients who may benefit from immunotherapy, targeted therapy, or chemotherapy alone. All relevant and available molecular and immunological targets in clinical practice for the systemic treatment of this disease are presented. Particular attention is given to possible future approaches, including circulating tumor DNA (ctDNA) for therapeutic monitoring, new targeting agents against molecular pathways such as fibroblast growth factor receptor (FGFR) and MET, chimeric antigen receptor (CAR)-T cells, and cancer vaccines. This review aims to provide a comprehensive understanding of current targets in advanced gastric cancer and to offer valuable insights into future directions of research and clinical practice in this challenging disease.

## 1. Introduction

Gastric cancer (GC) is the fifth most common cancer worldwide, characterized by low survival rates, aggressive nature, late-stage diagnosis, and limited therapeutic options [1]. As the third leading cause of cancer-related deaths, gastric cancer poses a significant global health issue [2]. Curative treatment in cases of early diagnosis involves surgery (total/subtotal gastrectomy) with lymphadenectomy, to which, given the high rate of recurrence (approximately 60%) [3], is added perioperative chemotherapy with the FLOT regimen [4,5]. In cases of advanced GC, the treatment is challenging because of its heterogeneity, which is associated with multiple factors from genomic to environmental levels, and molecular classifications are critical for guiding personalized therapies for GC [6]. The Cancer Genome Atlas network classified gastric cancer into four subtypes [7]: Epstein-Barr virus (EBV)-positive tumors, genomically stable (GS) tumors, microsatellite-instable (MSI) tumors, and tumors with chromosomal instability (CIN), using genomic and molecular platforms. Similarly, the Asian Cancer Research Group also analyzed gene expression and proposed four subtypes of molecular classification for gastric cancer: (a) microsatellite stable (MSS)/epithelial–mesenchymal transition (EMT), (b) MSI, (c) MSS–TP53-active, and (d) MSS–TP53-negative (e). In the early 21st century, fluoropyrimidine-based combined with platinum-based chemotherapy was the established first-line treatment for unresectable advanced gastric or gastroesophageal junction adenocarcinoma for a long time but has now given way to targeted drugs for genes and signaling pathways (f). Beyond targeting HER2, ongoing trials continue to unravel new molecular aberrations that could serve as potential therapeutic targets, such as MET, FGFR, and claudin 18.2 [8]. Moreover, the exploration of immune checkpoint inhibitors has opened a new era in cancer treatment [9]. Gastric cancer can be treated with immune checkpoint inhibitors alone or in combination with chemotherapy [9]. Other new treatment options for this disease are the incorporation of novel therapeutic modalities, such as antibody-drug conjugates (ADCs), cancer vaccines, and CAR-T cells [9]. Unfortunately, even despite advancements in diagnostic technologies and therapeutic strategies, the prognosis for patients with metastasizing GC remains poor, with a five-year survival rate below 10% [2]. This comprehensive review elucidates the current therapeutic targets, explores emerging perspectives in the management of this disease, and discusses the relevant clinical studies (summarized in Table 1), providing a critical synthesis of recent advances and future directions in the field.

## 2. Anti-Her2 Treatments

**Figure 1:**
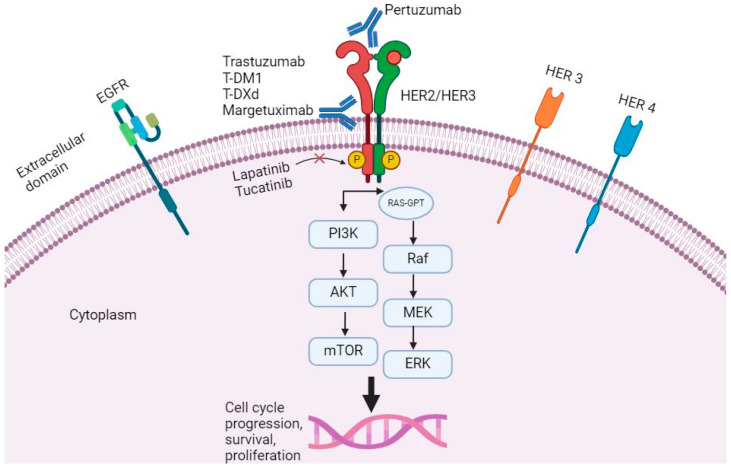
The figure illustrates the HER2 signaling pathways and the intracellular and extracellular binding sites of anti-HER2 antibodies and TKIs.

The role of anti-HER2 therapies, including checkpoint inhibitors and tyrosine kinase inhibitors (TKIs), in the treatment of metastatic GC has garnered significant interest due to their potential to improve patient outcomes (Figure 1) [10].

The landmark ToGA trial demonstrated that the anti-HER2 antibody trastuzumab significantly improves overall survival (OS) in HER2-positive advanced gastric and gastroesophageal junction adenocarcinomas. This trial established trastuzumab combined with chemotherapy as a standard first-line treatment for metastatic GC patients, with a median OS of 13.8 months for patients receiving trastuzumab in combination with chemotherapy, compared to 11.1 months for those receiving chemotherapy alone (hazard ratio (HR) = 0.74; 95% confidence interval (CI): 0.60–0.91). Additionally, the median progression-free survival (PFS) was 6.7 months versus 5.5 months (HR = 0.71, 95% CI: 0.59–0.85), and the objective response rate (ORR) was 47% versus 35%, respectively [11]. TKIs such as lapatinib and tucatinib have been extensively examined in HER2-positive metastatic gastric cancer [10]. Lapatinib, a reversible dual tyrosine kinase inhibitor targeting HER2 and EGFR, has shown improvements in PFS and OS when combined with chemotherapy. The TRIO-013/LOGiC trial reported an HR of 0.91 for OS (95% CI: 0.73–1.12) and an HR of 0.82 for PFS (95% CI: 0.67–1.00), although these results did not achieve statistical significance for OS [12]. Tucatinib is an orally administered small-molecule TKI that selectively inhibits HER2 tyrosine kinase activity with minimal inhibition of EGFR [13]. In preclinical studies, tucatinib combined with trastuzumab demonstrated significant antitumor activity in HER2-positive gastric cancer models [13]. Clinical trials have further investigated its efficacy and safety profile in various settings. The combination of tucatinib with trastuzumab and oxaliplatin-based chemotherapy, or pembrolizumab, is being evaluated in an ongoing phase 1b/2 trial for HER2-positive gastrointestinal cancers. Preliminary results suggest significant antitumor activity with manageable toxicities [14]. A phase 3 trial, MOUNTAINEER-02, is assessing tucatinib in combination with trastuzumab, ramucirumab, and paclitaxel for second-line treatment of HER2-positive metastatic gastric or gastroesophageal junction adenocarcinoma. This study aims to confirm the efficacy of tucatinib in improving OS and PFS compared to standard treatments [15]. Recent advancements highlight the potential of trastuzumab deruxtecan (T-DXd), an antibody-drug conjugate, which has shown significant efficacy in later-line therapy for HER2-positive metastasizing gastric cancer. The DESTINY-Gastric01 study demonstrated that T-DXd significantly improved median OS (12.5 months vs. 8.4 months; HR: 0.59, 95% CI: 0.39–0.88) and PFS (5.6 vs. 3.5 months; HR: 0.47, 95% CI: 0.31–0.71) compared to physician’s choice chemotherapy [16]. The DESTINY-Gastric02 trial evaluated T-DXd in patients with HER2-positive advanced gastric cancer who progressed after trastuzumab treatment, showing an overall response rate (ORR) of 38% and median PFS and OS of 5.6 and 12.1 months, respectively [17]. Additionally, the DESTINY-Gastric03 trial is exploring T-DXd combined with other therapies for both previously treated and untreated HER2-positive and HER2-low gastric cancer cases [18]. An ongoing phase 3 study, DESTINY-Gastric04, is comparing T-DXd with ramucirumab plus paclitaxel for similar patients [19]. All these studies aim to refine the therapeutic potential of T-DXd in earlier treatment lines and in combination with immunotherapies. Margetuximab is an Fc-engineered HER2 monoclonal antibody designed to enhance its binding affinity to CD16A and reduce its affinity to CD32B, thus improving antibody-dependent cellular cytotoxicity and other immune responses [20]. A phase 1b trial in HER2-positive advanced solid tumors, including gastric cancer, showed that margetuximab exhibited greater cytotoxicity than trastuzumab, with an ORR of 12% and a median PFS of 14 weeks [21]. In another phase 1b/2 study involving patients with relapsed or refractory HER2-positive gastroesophageal adenocarcinoma, margetuximab combined with pembrolizumab demonstrated a median OS of 12.5 months and an ORR of 44% in patients with high HER2 expression and PD-L1 positivity [22]. The MAHOGANY study is evaluating margetuximab with retifanlimab as first-line therapy in HER2-positive/PD-L1-positive unresectable or metastatic gastroesophageal adenocarcinoma, showing promising antitumor activity and manageable toxicities [23].

Zanidatamab, a bispecific HER2-targeted antibody, binds to two distinct extracellular domains of HER2 and has shown efficacy in various HER2-expressing cancers [24]. Zanidatamab promotes receptor clustering and internalization, inhibits tumor cell proliferation, and activates antibody-dependent cellular cytotoxicity (ADCC) [24]. A phase 1 trial demonstrated clinically meaningful antineoplastic effects in HER2-positive cancers, including advanced gastric cancer [25]. In a phase 2 study, zanidatamab combined with standard chemotherapy (CAPOX, mFOLFOX6, or FP) as first-line treatment for HER2-positive advanced gastric/gastroesophageal junction cancer showed a confirmed ORR of 79%, with a median response duration of 20.4 months, PFS of 12.5 months, and OS not yet reached [26].

Recent progress has been made in combining trastuzumab with immune checkpoint inhibitors, showing promise for HER2-positive gastric cancer, with ongoing trials expected to provide further insights. Trastuzumab has shown a positive effect on antitumor immune responses in HER2-positive gastric cancer, promoting the uptake of HER2 by dendritic cells, inducing cytotoxic T-lymphocytes [27], and upregulating PD-L1 expression, making PD-1/PD-L1 inhibition a promising treatment strategy [28]. Clinical trials combining pembrolizumab, a PD-1 antibody, with chemotherapy and trastuzumab have shown higher ORR and tumor burden reduction [29,30]. The ongoing KEYNOTE-811 phase 3 trial is evaluating the efficacy of chemotherapy plus trastuzumab with pembrolizumab vs. chemotherapy plus trastuzumab with placebo for untreated HER2-positive gastric cancer [29]. The interim analysis showed a significant improvement in PFS for the pembrolizumab group (10.0 months vs. 8.1 months; HR: 0.72; 95% CI: 0.60–0.87; *p* = 0.0002) and a higher ORR (72.6% vs. 59.8%). However, OS was not significantly longer [29]. Another randomized phase 2 study compared trastuzumab plus nivolumab with ipilimumab versus trastuzumab plus FOLFOX in untreated HER2-positive gastric cancer, showing that the FOLFOX group had a higher 12-month OS rate (70% vs. 57%) [30].

## 3. Targeting Pd-L1 and MSI: The Role of Immune Checkpoint Inhibitors

PD-1 and PD-L1 are paired molecular compounds expressed on the surface of T-cells and cancer cells, respectively [31]. Their interaction causes immune evasion, suppression of immune killing functions, and activation of tumor growth. Therefore, PD-1 and PD-L1 blockade inhibits the interaction between the two antigens and reactivates the downstream immune antitumoral function [32]. PD-L1 expression is predictive of the efficacy of anti-PD-1 treatment. It can be evaluated with immunohistochemistry either by the combined positive score (CPS) or tumor proportion score (TPS) [32]. CPS is calculated based on the number of PD-L1-positive cancer and mononuclear inflammatory cells in relation to total viable tumor cells, while TPS considers the number of PD-L1-positive neoplastic cells in relation to the total number of viable tumor cells [32]. A CPS/TPS score ≥ 1 indicates positive PD-L1 expression, with a prevalence of PD-L1 ≥ 1 ranging between 50 and 60% of total gastric cancer cases [2]. Response rates and OS are also higher in gastric cancer patients with mismatch repair deficient or microsatellite-instable (MSI-H) tumors treated with immunotherapy. Indeed, cancer-agnostic indications with anti-PD-1 therapy are available for MSI-H patients [3].

Combinations of nivolumab plus chemotherapy or plus ipilimumab and pembrolizumab plus chemotherapy are available as first-line treatment options in Western countries [33]. In particular, the efficacy of immune-checkpoint inhibitors in this scenario was evaluated in the randomized phase III trials for HER-2 negative gastric, esophageal, and gastroesophageal junction (GEJ) tumors: Checkmate 648 [34], Keynote 590 [35], and Keynote 859 [36].

In the Checkmate 649 trial, previously untreated patients with gastroesophageal adenocarcinoma were randomly assigned to either nivolumab plus chemotherapy, nivolumab plus ipilimumab, or chemotherapy alone. Nivolumab plus chemotherapy achieved an improvement in OS compared to chemotherapy in patients with PD-L1 CPS ≥ 5 (median OS: 14.4 vs. 11.1 months, HR 0.71) and all randomized patients (median OS: 13.8 vs. 11.6 months, HR 0.79) [37]. Survival benefit did not achieve statistical significance in tumors with CPS < 5, leading the EMA to restrict approval of combination therapy to the CPS ≥ 5 population alone [2]. Results were confirmed in a 3-year follow-up update, with an HR for OS of 0.70 and 21% of patients alive at 36 months in the experimental arm compared to the control arm [18].

The Keynote 590 trial randomized patients with previously untreated metastatic esophageal and GEJ tumors (Siewert I) to receive either standard first-line chemotherapy with cisplatin and 5-fluorouracil alone or in combination with the anti-PD-1 agent pembrolizumab. Most patients had esophageal squamous carcinoma (ESCC), and 53% were from Asian countries. The trial showed a 5-month OS improvement in ESCC with PD-L1 CPS ≥ 10 (13.9 months vs. 8.8 months, HR 0.57). Moreover, an OS advantage was also recorded for the whole ESCC population (HR 0.72), PD-L1 CPS of 10 or more with HR 0.62, and in all randomized patients (HR 0.73). No benefit was seen from the addition of pembrolizumab in the PD-L1 CPS < 10 population. Similarly, pembrolizumab plus chemotherapy was superior to placebo plus chemotherapy for PFS in all subgroups [35]. The trial led to the regulatory approval of pembrolizumab in combination with platinum and fluoropyrimidine-based chemotherapy, with European Medicines Agency (EMA) indications restricted to the PD-L1 CPS ≥ 10 [2]. Recently, results were confirmed in a 5-year follow-up update, with a median OS of 12.3 months for the experimental arm vs. 9.8 months for the control arm (HR 0.72), while 5-year OS rates were 10.6 and 3%, respectively [36].

The Keynote 859 trial included patients with previously untreated metastatic gastric and GEJ tumors to receive a combination of cisplatin and 5-fluorouracil (or capecitabine and oxaliplatin) in association with pembrolizumab or placebo. Patients were homogenously distributed among different geographical regions (33% Asian, 25% from Western Europe, Israel, North America, and Australia, and 41% from the rest of the world). Median OS was longer in the pembrolizumab arm than in the placebo arm (12.9 months vs. 11.5 months, HR 0.78, *p* < 0.0001) in participants with a PD-L1 CPS of 1 or higher (HR 0.74) and in participants with a PD-L1 CPS of 10 or higher (HR 0.65). Participants in the pembrolizumab plus chemotherapy group had significantly improved PFS and ORR compared with the placebo plus chemotherapy group as well [36].

For the second-line treatment, the phase II Keynote-158 trial included 24 patients with gastric cancer [38]. Pembrolizumab monotherapy was highly active in pretreated MSI-H gastric cancer patients, achieving an ORR of 45.8% and a median PFS of 11 months, while median OS and median duration of response were not reached [38]. Therefore, in this subgroup of patients not pretreated with a combination of chemotherapy and immunotherapy, pembrolizumab should be the preferred treatment option [2].

## 4. Anti-VEGF Treatments

Angiogenesis is a fundamental stage and prerequisite for the growth of cancer and metastasis [39]. It is well established that vascular endothelial growth factor (VEGF) plays a pivotal role in angiogenetic processes by stimulating the proliferation, migration, and survival of microvascular endothelial cells and inducing vascular permeability [40,41,42]. Besides VEGF, tumor cells and stromal cells produce various further angiogenic factors in GC; of particular interest are interleukin-8 (IL-8) and platelet-derived endothelial cell growth factor (PD-ECGF) [43]. Therefore, the inhibition of angiogenesis has also received considerable attention as a therapeutic option in GC [44,45,46].

Examples of drugs that interfere with the angiogenesis process include monoclonal anti-VEGF antibodies, VEGF-Trap, and small-molecule tyrosine kinase inhibitors [39,40,41].

Ramucirumab is an intravenous, fully human IgG1 monoclonal antibody that specifically binds to VEGF-receptor 2, inhibiting its interaction with ligands VEGF-A, VEGF-C, and VEGF-D [47,48,49,50]. In 2014, the RAINBOW trial (comparing ramucirumab plus paclitaxel to placebo plus paclitaxel) and the REGARD trial (examining ramucirumab monotherapy) demonstrated the effectiveness of this anti-VEGF therapy in patients with previously treated advanced gastric or gastro–oesophageal junction adenocarcinoma [51,52]. These trials showed a significant improvement in median overall survival: 9.6 vs. 7.4 months in the RAINBOW trial and 5.2 vs. 3.8 months in the REGARD trial. As a result, ramucirumab became the first targeted angiogenesis therapy for advanced gastric cancer. However, combining ramucirumab with an irinotecan-based regimen has not yielded successful results as a second-line treatment for advanced gastric cancer [53]. Currently, combination therapy with weekly paclitaxel and ramucirumab is recommended as the standard second-line chemotherapy for these patients [2,54]. Ramucirumab did not improve survival in previously untreated patients with advanced GC, regardless of backbone chemotherapy regimens or administration schedules. A phase III trial (RAINFALL) and two phase II trials (JVBT and RAINSTORM) were conducted to elucidate the add-on effect of ramucirumab with a platinum doublet (fluoropyrimidine plus CDDP, mFOLFOX6, and SOX, respectively) as first-line chemotherapy for patients with advanced GC, but no survival benefit from the addition of ramucirumab to chemotherapy was identified [55,56,57].

Trifluridine/tipiracil (Lonsurf) is a fixed-dose combination tablet comprising trifluridine, an antineoplastic nucleoside analog, and tipiracil, a thymidine phosphorylase inhibitor [58]. Trifluridine inhibits cell proliferation by direct insertion into the DNA after phosphorylation, leading to DNA dysfunction and cell death. Tipiracil interacts with the metabolization of trifluridine through inhibition of thymidine phosphorylase, thus allowing high systemic exposure to trifluridine [58,59,60].

The use of oral trifuridine/tipiracil 35 mg/m^2^ twice daily on days 1–5 and 8–12 of each 28-day cycle is approved in patients with advanced GC who had previously been treated with at least two chemotherapy regimens for advanced disease. This is based on the results from the randomized phase III TAGS trial, which demonstrated increased OS against placebo (median, 5.7 vs. 3.6 months) with acceptable (mainly hematologic) toxicity [61].

Recently, Lonsurf, in combination with anti-angiogenic drugs (i.e., ramucirumab and bevacizumab), has been suggested as a promising late-line treatment regimen for advanced GC [62,63,64]. A phase II study on the combination of Lonsurfith and ramucirumab in GC revealed a disease control rate of 77% [64]. In addition, a phase III study evaluating the effect of sustained use of ramucirumab beyond disease progression in GC is ongoing [63].

Fruquintinib is a novel, highly selective, and potent oral inhibitor of VEGFR 1, 2, and 3. The agent has improved kinase selectivity, resulting in decreased off-target toxicities and increased tolerability [65,66]. Fruquintinib is already approved for the treatment of metastatic colorectal cancer, based on the results of the FRESCO [65] and FRESCO-2 phase III trials [66], conducted, respectively, in China and worldwide. In both trials, the new drug showed a significant improvement in OS. Common side effects include hypertension, proteinuria, and hand-foot syndrome, but these are generally less severe than the adverse effects associated with conventional chemotherapy.

In the randomized, double-blind, placebo-controlled, phase III FRUTIGA trial, the efficacy and safety of fruquintinib plus paclitaxel vs. paclitaxel alone were investigated in a total of 703 patients with advanced gastric/GEJ adenocarcinoma after disease progression on fluoropyrimidine- or platinum-based first-line chemotherapy [67]. Once enrolled, patients were randomized in a 1:1 ratio to receive fruquintinib at a dose of 4 mg once daily for 3 weeks on/1 week off or a matching placebo given orally, plus paclitaxel at a dose of 80 mg/m^2^ via intravenous infusion on days 1/8/15 per cycle in 4-week cycles until progressive disease or intolerable toxicity. The study met the predefined criteria for PFS, demonstrating a significant improvement with fruquintinib plus paclitaxel vs. placebo plus paclitaxel (median = 5.6 vs. 2.7 months, HR = 0.57, *p* < 0.0001). ORR in the fruquintinib group was nearly two-fold higher than the placebo group (42.5% vs. 22.4%, respectively). Fruquintinib plus paclitaxel demonstrated a trend for OS benefit, but this difference was not statistically significant (median = 9.6 vs. 8.4 months, HR = 0.96, *p* = 0.6064). Post hoc analyses adjusting for confounding effects supported the OS benefit of fruquintinib plus paclitaxel, with an HR ranging from 0.73 to 0.91 [67]. In addition, the ORR was significantly higher among patients in the fruquintinib and paclitaxel groups compared with the placebo group [67]. Interestingly, median PFS was extended even more among non-diffuse gastric or gastroesophageal adenocarcinoma patients with lymph node metastases (6.1 months in the fruquintinib group vs. 2.7 months in the placebo group, *p* < 0.0001), and OS also showed a nominally statistically significant improvement (9.6 vs. 7.9 months, *p* = 0.0233) [68]. Hence, targeting VEGFRs offers a strategic approach to metastasizing GC, but more trials are needed to test their activity in combination with immunotherapy and in a first-line setting.

## 5. Targeting Claudin 18.2

Claudin 18.2 (CLDN18.2), a tight junction protein, has emerged as a promising therapeutic target in metastatic GC [69]. The restricted expression in normal tissues and prevalent overexpression in GC make it an attractive candidate for targeted therapy [69]. In fact, it is uniquely found in the gastric mucosa and is not present in other healthy tissues. During malignant transformation, CLDN18.2 expression may persist in various tumor tissues, including gastric/gastroesophageal junction (G/GEJ) cancers, particularly in diffuse-type GC. The reported prevalence of CLDN18.2 overexpression in GC varies significantly, ranging from 14.1% to 72% [69].

Zolbetuximab is a chimeric IgG1 monoclonal antibody that targets CLDN18.2, triggering antibody-dependent and complement-dependent cytotoxicity [70]. Zolbetuximab has shown significant potential as a therapeutic target in GC. In the phase II MONO study, zolbetuximab as a single agent achieved an ORR of 9% and a disease control rate of 23% in 43 patients with previously treated esophageal or G/GEJ cancers [70]. The pivotal FAST study demonstrated significant survival benefits with zolbetuximab [71]. In this phase IIb trial, patients with Claudin 18.2-positive, HER2-negative advanced GC were randomized to receive zolbetuximab plus EOX (epirubicin, oxaliplatin, and capecitabine) versus EOX alone [71]. The zolbetuximab group showed a median OS of 13.2 months vs. 8.4 months in the control group (HR = 0.55, *p* < 0.001), highlighting its potential as a first-line treatment. Subgroup analysis indicated a correlation between moderate-to-strong CLDN18.2 expression and improved OS rates [72]. In the phase III SPOTLIGHT trial, zolbetuximab plus mFOLFOX6 significantly increased median PFS (10.61 vs. 8.67 months, HR 0.751, *p* = 0.0066) and median OS (18.23 vs. 15.54 months, HR 0.750, *p* = 0.0053) in patients with CLDN18.2-positive and HER2-negative advanced G/GEJ cancer [73]. Another phase III trial (GLOW study) is assessing zolbetuximab combined with CAPOX as a first-line treatment for patients with CLDN18.2-positive, HER2-negative, locally advanced unresectable, or advanced gastric or GEJ cancer [74]. This study revealed that zolbetuximab plus CAPOX significantly improved median PFS (8.21 vs. 6.80 months, HR 0.687, *p* = 0.0007) and median OS (14.39 vs. 12.16 months, HR 0.771, *p* = 0.0118) compared to placebo plus CAPOX [75]. Additionally, zolbetuximab is being evaluated in combination with immunotherapy for CLDN18.2-positive advanced gastric or GEJ cancer in the ILUSTRO study [76]. In several clinical trials, zolbetuximab was shown to be generally well-tolerated, with most adverse events being manageable. Common side effects include nausea, vomiting, and neutropenia [70,71,72,73].

Another promising treatment strategy involves CLDN18.2-specific chimeric antigen receptor (CAR) T-cells [77,78]. Currently, several new drugs targeting Claudin 18.2, such as Claudin 18.2 bispecific antibodies (Claudin 18.2/CD3, Claudin 18.2/PD-L1) and ADCs, are in development [79]. Although these drugs have not yet received clinical approval, some have shown promising preclinical results and are currently being extensively studied in various clinical trials [79]. However, resistance mechanisms, including claudin 18.2 heterogeneity and antigen loss, are emerging as challenges [79]. Understanding these mechanisms is crucial for optimizing treatment strategies and developing next-generation therapies.

## 6. New Frontiers in Metastatic Gastric Cancer

### 6.1. Potential Applications of ctDNA

**Figure 2:**
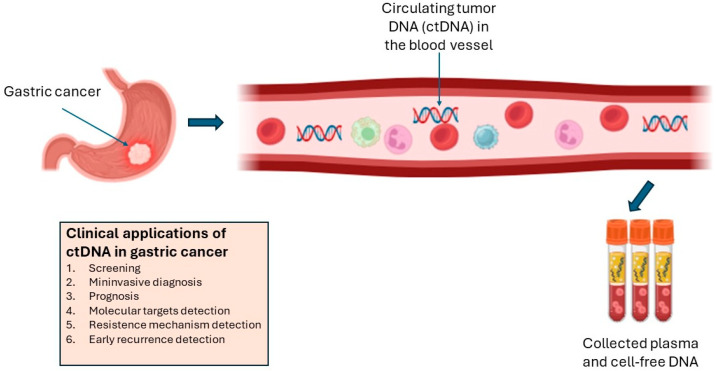
Clinical applications of circulating tumor DNA (ctDNA) in metastatic gastric cancer and its isolation from blood vessel.

Circulating tumor DNAs (ctDNAs) are small DNA fragments released by cancer via apoptosis, necrosis, or active release into the bloodstream (Figure 2) [75,80]. ctDNAs have an average length of 143–145 base pairs, and they are generally smaller than non-tumor plasma DNAs [81]. In the last few years, ctDNAs have emerged as a promising tool in cancer management, including GC [74,82,83]. In fact, it could be useful both in early diagnosis, supporting the detection of minimal residual disease after curative surgery, and in the metastatic setting for treatment decision choice and therapeutic monitoring [84,85,86,87,88,89,90]. ctDNAs are released by different cancerous subclones and, therefore, are more representative of molecular tumor heterogeneity compared to biopsy analysis [80,81]. ctDNA analysis provides a comprehensive picture of cancer by capturing all mutations, insertions, deletions, rearrangements, copy number variations, and methylations [84,85,86,87,88,89,90]. Despite these advantages, the integration of ctDNA-based approaches into routine clinical practice remains limited [87]. While ctDNA analysis holds high potential, its widespread adoption requires further validation and standardization [84,85,86,87,88,89,90]. Nonetheless, recent studies have underscored the clinical utility of ctDNA as a prognostic biomarker in advanced GC [87,88,89,90]. In a large study based on more than 1500 metastatic GC patients, it was revealed that patients who experienced a >50% decline in ctDNA after treatment had a significantly longer median OS compared to those with <50% decline. This was observed in patients treated with chemotherapy as well as in patients receiving immunotherapy [91]. In summary, dynamic changes in ctDNA levels can serve as a reliable indicator of treatment response and disease progression [91,92].

In advanced GC, an association between ctDNA-detected chromosomal instability and response to chemotherapy has been described [93]. One study observed a reduction in the copy number instability score using ctDNA post-treatment, correlating with enhanced treatment response, particularly among patients exhibiting chromosomal instability (ORR 59%) compared to those with stability (ORR 32%) [93]. However, contradictory results were reported by other studies, necessitating further prospective trials for confirmation [92,93].

Concordance assessments between ctDNA and tissue-based HER2 amplification revealed variable results [92,94]. ctDNA has been shown to predict and monitor treatment response in HER2-positive GC [92,94,95]. Notably, in HER2-positive metastatic GC, the baseline ctDNA ERBB2 copy number correlated with treatment response and underlined prognostic utility [92,94,95]. Furthermore, ctDNA can be used to identify resistance mechanisms to anti-HER2 therapies [92,94,95].

With the advent of chemo-immunotherapy as the first-line standard in advanced GC, the assessment of PD-L1 status became mandatory [96]. Nevertheless, PD-L1 determination alone should be used with caution for clinical decision-making. Several studies have established ctDNA dynamics as an additional predictor for response to anti-PD1 inhibitors [92,96,97]. Furthermore, ctDNA based mutation analysis revealed specific mutations in TGFBR2, RHOA, and PREX2 are connected with worse outcomes of immunotherapeutic treatments [92,96,97]. In addition, it was possible to prove a correlation between mutations in FGFR4, MET, CEBPA, and KMT2B and more immune-related adverse events in GC patients [92,96,97]. Exploration of ctDNA-based Epstein-Barr virus (EBV) detection for EBV-associated GC highlighted challenges in concordance with tissue analysis but nevertheless suggested a high potential as a response predictor [98]. Moreover, ctDNA analysis facilitates the detection of FGFR2 overexpression, guiding treatment decisions and providing a potential tool for monitoring the response to anti-FGFR drugs [99].

The umbrella VIKTORY trial showed the utility of ctDNA in identifying targetable alterations for personalized treatment in the context of metastatic GC [100]. This study also highlighted the potential of ctDNA monitoring to predict treatment response earlier than radiological assessments, especially in MET-mutated patients [100]. However, comprehensive validation through larger prospective studies is imperative to confirm these promising findings [101].

### 6.2. Promising Molecular Targets: FGFR and MET

FGFR2 alterations are prevalent in both GC and esophagogastric junction adenocarcinoma, ranging from 9% to 61% of patients [6]. Therefore, several FGFR inhibitors have been explored in FGFR-overexpressing GC [6,102].

AZD4547, a pan-FGFR reversible tyrosine kinase inhibitor, was assessed in the phase II SHINE study but failed to improve median PFS compared to paclitaxel (1.8 months vs. 3.5 months, *p* = 0.9581), most possibly due to intratumoral heterogeneity [103].

Futibatinib, an irreversible FGFR isoform inhibitor, demonstrated promising activity in phase I studies, with notable responses in patients with FGFR2 amplification or fusion [104,105,106,107].

Bemarituzumab, a specific anti-FGFR2 monoclonal antibody, exhibited encouraging results in a phase I monotherapy study and the phase II FIGHT trial [108,109]. Although bemarituzumab did not significantly improve median PFS in combination with modified FOLFOX6 chemotherapy compared to placebo (9.5 vs. 7.5 months, *p* = 0.073), a post-hoc analysis revealed prolonged median OS (19.2 vs. 13.5 months, HR 0.60, 95% CI 0.38–0.94), particularly in patients with FGFR2b overexpression (HR 0.41, 95% CI 0.23–0.74) [109]. The ongoing phase III FORTITUDE-102 trial aims to further evaluate bemarituzumab in combination with chemotherapy and nivolumab in FGFR2b-overexpressed GC and GEJ [110].

The c-Mesenchymal–Epithelial Transition (c-MET) receptor, a member of the MET family of tyrosine kinase receptors, is activated by hepatocyte growth factor (HGF) [111]. Activation of the MET/HGF pathway has been implicated in promoting tumor invasiveness and predicting poor disease prognosis [111]. Despite the promising potential, clinical trials investigating the efficacy of MET inhibitors have reported contradictory results [111].

Onartuzumab, an anti-MET monoclonal antibody, failed to demonstrate improved clinical outcomes when combined with mFOLFOX6 in metastatic HER2-negative GC/GEJ cancers, both in the overall population and in the MET-positive subgroup [112].

Similarly, rilotumumab, a humanized monoclonal antibody targeting HGF, was investigated in phase III trials (RILOMET-1 and RILOMET-2) alongside chemotherapy for advanced MET-positive GC/GEJ cancers, but both studies were prematurely terminated due to an observed increase in mortality among participants receiving rilotumumab [113,114].

Furthermore, various selective and non-selective c-MET tyrosine kinase inhibitors, including tinvatinib, AMG 337, and foretinib, have been evaluated in clinical trials for MET-positive GC, yet none have shown significant clinical benefit [115,116,117]. These findings underscore the complexities of targeting the MET/HGF pathway in oncological therapy and highlight the need for further research to elucidate optimal treatment strategies [115,116,117].

### 6.3. Role of Homologous Recombination Deficiency

In several cancers, the DNA damage response pathway plays a crucial role, particularly in tumors exhibiting homologous recombination deficiency (HRD) [118]. This deficiency enhances the effectiveness of chemotherapy using platinum salts and inhibitors of poly(ADP-ribose) polymerase (PARP) [119]. BRCA mutations, whether germline or somatic, are commonly used as biomarkers in this treatment strategy [120]. Additionally, mutations in genes such as ATM, PALB2, and RAD51 are associated with HRD [118,119,120]. In GC, approximately 7–12% of cases exhibit mutations in genes related to HRD, with ATM being the most frequently mutated gene [121,122,123]. The phase III GOLD trial did not show a significant survival advantage from the addition of olaparib to taxane in the second-line treatment of advanced GC [124]. Nonetheless, subsequent translational analyses, including next-generation sequencing of 15 HRD-related genes, demonstrated improved outcomes in the subgroup with ATM mutations [125]. Ongoing clinical trials are exploring the use of anti-PARP agents in maintenance therapy after platinum-based chemotherapy, either alone or in combination with anti-angiogenic antibodies and immune checkpoint inhibitors [125,126].

### 6.4. CAR-T Cells

Cancer immunotherapy is witnessing a paradigm shift with the increasing utilization of immune-checkpoint inhibitors and CAR-T cells [127,128,129]. CAR T-cell therapy involves the genetic modification of T-cells to express engineered receptors that are tailored to recognize and target specific antigens present in cancer cells [127,128,129]. This genetic alteration activates T-cells, empowering them to mobilize the immune system for the identification and eradication of tumor cells [127,128,129]. CAR-T cells offer a viable therapeutic avenue for advanced GC by exhibiting promising efficacy in targeting biomarkers such as CLDN 18.2, HER2, mucin 1, natural killer receptor group 2, and mesothelin [128,129,130]. Moreover, CAR T-cell therapy could be able to overcome the problem of multidrug resistance [128,129]. Research focusing on HER2 CAR T-cell therapy has demonstrated compelling efficacy in addressing advanced GC [130]. Using CLDN18.2 CAR-T cells customized for CLDN18.2-positive individuals, they have exhibited remarkable antitumor activity [78]. It is noteworthy to mention here two preclinical CART-T cell approaches. Firstly, B7-H3 is often overexpressed in the tumor tissues of advanced GC patients and is closely related to disease progression [131]. CAR-T cells specifically directed against B7-H3 revealed strong antitumor efficacy and cytotoxicity against GC cells [131]. Secondly, CDH17 is frequently expressed in GC cells and plays a pivotal role in calcium-dependent adhesion switching and Wnt signaling pathways [132]. Preclinical studies in murine models showed the robust efficacy of CDH17 CAR T-cell therapy against advanced GC while sparing normal gastrointestinal epithelial cells from significant toxicity [133].

### 6.5. Cancer Vaccines

In the last few years, tumor vaccines have emerged as focal points of research [134]. There are four types of them: cell-based, protein- or peptide-based, or gene-based (DNA/RNA), primarily leveraging dendritic cells as their main adjuvants [134,135]. Cancer vaccines aim to overcome the immune suppression induced by tumors, boost immunogenicity, activate the patient’s immune system, and trigger both cellular and humoral immune reactions to cancer [134,135]. Research into vaccines targeting HER2 expression in neoplastic cells has led to significant advancements, indicating potential benefits for treating advanced breast cancer and potentially other solid malignancies with high HER2 expression [136,137]. One example is the B-cell epitope vaccine called IMU-131/HER-Vaxx, which comprises three fused B-cell epitopes from the HER2 extracellular domain coupled with CRM197 and Montanide as an adjuvant [78]. The phase II HERIZON trial was randomized to either HER-Vaxx plus standard chemotherapy or standard chemotherapy alone in patients with HER2/neu overexpressing metastasizing or advanced gastric/GEJ adenocarcinoma who were naïve to HER2 therapy [138]. The primary endpoint was OS. HER-Vaxx plus chemotherapy-treated patients received a 50 µg dose of HER-Vaxx by intra-muscular injection at days 0, 14, 35, 77, and every 63 days until disease progression. Both groups received chemotherapy starting at day 0 and then every 21 days for a maximum of 6 cycles or until disease progression. Standard chemotherapy consisted of cisplatin and 5FU, or capecitabine or oxaliplatin together with capecitabine. A total of 36 patients were randomized (19 treated with HER-Vaxx plus chemotherapy and 17 with chemotherapy alone). Analysis showed a 42% survival benefit for patients treated with HER-Vaxx plus chemotherapy compared to chemotherapy alone. The median OS for patients receiving HER-Vaxx plus chemotherapy was 13.9 (7.5, 14.3) months, compared to 8.3 (6.0, 9.6) months in patients treated with chemotherapy alone. The results of this phase II trial demonstrate that in patients with HER2 overexpressing gastric/GEJ cancer, active HER2 immunization with HER-Vaxx is safe and provides relevant clinical benefit over standard chemotherapy [138]. Dendritic cells are recognized as key players in the immune system, acting as antigen-presenting cells that stimulate and control the body’s adaptive immune response by presenting antigens to T-cells [78,137,138]. Specifically, dendritic cell vaccines are tailored to target tumor-specific neo-antigens, offering a personalized approach that has shown success in several clinical trials, making them a focal point of current research [139,140,141]. Dendritic cells fused with GC cells or loaded either with peptides or RNAs can effectively boost the immune response in patients [139,140,141]. mRNA vaccines show enhanced efficacy as well as rapid immune activation [142] and represent a valuable alternative to dendritic cell-based vaccinations [139,140,141]. Combinations of these vaccines with chemotherapeutic agents (e.g., cisplatin, 5-fluorouracil) have been tested in clinical trials and showed promising outcomes [9,134]. However, targeting GC by vaccines provides a formidable new treatment option, but it is limited due to antigenic shifts and immune evasion [142]. Further research is needed to tackle these obstacles.

## 7. Conclusions and Future Perspectives

The landscape of metastatic GC treatment is rapidly evolving, led by significant results from clinical trials focusing on molecular and immunological targets (summarized in Table 1). Future diagnostic and therapeutic approaches are focusing on personalized medicine, leveraging the potential of immunotherapy, including immune checkpoint inhibitors and CAR T-cell therapy.

Moreover, the integration of ctDNA analysis into clinical practice holds the potential for early diagnosis, treatment decision-making, and therapeutic monitoring in mGC.

The exploration of promising molecular targets, such as FGFR and MET pathways, offers new strategies for targeting metastatic GC. Although initial clinical trials have shown mixed results, ongoing research aims to elucidate optimal approaches and identify biomarkers for patient selection, with the aim of further personalizing the GC treatment.

Cancer vaccines, in particular dendritic cell and mRNA vaccines, offer a personalized approach to fueling the immune response against tumors. Combining mRNA vaccines with chemotherapeutic agents showed promising outcomes, although challenges such as immune evasion and antigenic shifts pose significant obstacles to their use in metastatic GC treatment.

Future research and clinical trials are needed to validate current and new biomarkers and to optimize the combination of all the therapeutic strategies in order to address the complexities of this challenging disease.

## Figures and Tables

**Table 1:** Overview of the most significant clinical trials in metastatic gastric cancer.

Study Name	Clinical Trial Number	Phase	Agent Tested	Outcome
ToGA	NCT01041404	3	Trastuzumab	Improved OS in HER2-positive metastatic gastric and gastroesophageal junction adenocarcinoma resulted in establishing trastuzumab combined with chemotherapy as a standard first-line treatment for metastatic gastric cancer patients.
TRIO-013/LOGiC	NCT00680901	3	Lapatinib	Lapatinib has been shown to improve PFS and OS when combined with chemotherapy.
SGNTUC-024	NCT04430738	1b/2	Combination of tucatinib with trastuzumab and oxaliplatin-based chemotherapy or pembrolizumab	Ongoing study—no results reported up to now.
MOUNTAINEER-02	NCT04499924	3	Combination of tucatinib with trastuzumab, ramucirumab, and paclitaxel for second-line treatment of HER2-positive metastatic gastric or gastroesophageal junction adenocarcinoma	Ongoing study—no results reported up to now.
DESTINY-Gastric01	NCT03329690	2	Trastuzumab deruxtecan	Improved median OS and PFS in HER2-positive metastatic gastric cancer.
DESTINY-Gastric02	NCT04014075		Trastuzumab deruxtecan	Improved median OS and PFS in HER2-positive advanced gastric cancer who progressed after trastuzumab treatment.
DESTINY-Gastric03	CT04379596		Trastuzumab deruxtecan combined with immunotherapies	Ongoing study—no results reported up to now.
DESTINY-Gastric04	NCT04704934	3	Comparing trastuzumab deruxtecan with ramucirumab plus paclitaxel	Ongoing study—no results reported up to now.
Safety study of MGAH22 in HER2-positive carcinomas	NCT01148849	1b	Margetuximab	Margetuximab exhibited greater cytotoxicity than trastuzumab in HER2-positive advanced gastric cancer.
CP-MGAH22-0	NCT02689284	1b/2	Margetuximab combined with pembrolizumab	Proof of concept of synergistic antitumor activity with the combination of an anti-HER2 agent along with anti-PD-1 checkpoint blockade in patients with high HER2 expression and PD-L1 positivity.
MAHOGANY	NCT04082364	2/3	Margetuximab with retifanlimab	Promising antitumor activity and manageable toxicities in HER2-positive/PD-L1-positive unresectable or metastatic gastroesophageal adenocarcinoma.
Trial of ZW25 in Patients with advanced HER2-expressing cancers	NCT02892123	1	Zanidatamab	Clinically meaningful antitumor effects of HER2-positive advanced gastric cancer.
First-Line Zanidatamab plus Chemotherapy for HER2-expressing metastatic gastroesophageal adenocarcinoma	NCT03929666	2	Zanidatamab combined with standard chemotherapy	Improved PFS in HER2-positive metastatic gastric/gastroesophageal junction cancer; OS not yet reached.
Checkmate 649	NCT02872116	3	Nivolumab plus chemotherapy, nivolumab plus ipilimumab versus chemotherapy alone	Nivolumab plus chemotherapy achieved an improvement in OS compared to chemotherapy in patients with PD-L1 CPS ≥ 5. Survival benefit did not achieve statistical significance in tumors with CPS < 5, leading to restricted approval of combination therapy to CPS ≥ 5 population in HER2-negative gastric or gastro–oesophageal junction adenocarcinoma.
INTEGA	NCT03409848	2	trastuzumab plus nivolumab with ipilimumab versus trastuzumab plus FOLFOX	The FOLFOX group had a higher 12-month OS in untreated HER2-positive gastric cancer.
Keynote 590	NCC201807010	2	standard first-line chemotherapy with cisplatin and 5-fluorouracil alone or in combination with pembrolizumab	Improved OS was seen in previously untreated metastatic esophageal and gastroesophageal junction tumors with PD-L1 CPS ≥ 10, but no benefit was seen for the addition of pembrolizumab in the PD-L1 CPS < 10 population. The trial led to the regulatory approval of pembrolizumab in combination with platinum and fluoropyrimidine-based chemotherapy restricted to PD-L1 CPS ≥ 10.
Keynote 859	NCT03675737	3	Combination of cisplatin and 5-fluorouracil (or capecitabine and oxaliplatin) with pembrolizumab	Participants with untreated metastatic gastric and gastroesophageal junction tumors in the pembrolizumab plus chemotherapy group had significantly improved PFS and ORR.
Keynote-158	NCT02628067	2	Pembrolizumab	Pembrolizumab monotherapy was highly active in pretreated microsatellite-high gastric cancer patients.
RAINBOW	NCT01170663	3	Ramucirumab plus paclitaxel	Significant improvement in median OS in previously treated advanced gastric or gastro–oesophageal junction adenocarcinoma. Based on these results, ramucirumab became the first targeted therapy for angiogenesis in patients with advanced gastric cancer.
REGARD	NCT00917384	3	Ramucirumab	Significant improvement in median OS in previously treated advanced gastric or gastro–oesophageal junction adenocarcinoma. Based on these results, ramucirumab became the first targeted therapy for angiogenesis in patients with advanced gastric cancer.
RAMIRIS	NCT03081143	2	Ramucirumab plus FOLFIRI versus paclitaxel plus ramucirumab	Combination therapy with weekly paclitaxel and ramucirumab is recommended as the standard second-line chemotherapy for patients with advanced gastric cancer.
RAINFALL	NCT02314117	3	Ramucirumab plus cisplatin and fluoropyrimidine	No survival benefit from the addition of ramucirumab to chemotherapy as first-line therapy was identified in previously untreated patients with advanced gastric cancer.
RINDBeRG	UMIN000023065	3	Ramucirumab plus irinotecan in third or more line beyond progression after ramucirumab for advanced gastric cancer	Ongoing study—no results reported up to now.
RAINSTORM	NCT02539225	2	Ramucirumab plus S-1 and oxaliplatin	No survival benefit from the addition of ramucirumab to chemotherapy was identified in previously untreated patients with advanced gastric cancer.
TAGS	NCT02500043	3	Lonsurf	Increased OS against placebo with acceptable toxicity in patients with advanced gastric cancer who had previously been treated with at least two chemotherapy regimens for advanced disease.
Enhanced efficacy of anti-VEGFR2/taxane therapy after progression on immune checkpoint inhibition	JapicCTI-194596	2	Lonsurf in combination with anti-angiogenic drugs (i.e., ramucirumab and bevacizumab)	Acceptable safety profile and clinical activity in patients with previously treated advanced gastric cancer regardless of previous ramucirumab exposure.
FRUTIGA	NCT03223376	3	Fruquintinib plus paclitaxel versus paclitaxel alone	Improved PFS and ORR with fruquintinib plus paclitaxel in patients with advanced gastric and gastroesophageal junction adenocarcinoma after disease progression on fluoro-pyrimidine- or platinum-based first-line chemotherapy.
MONO	NCT01197885	2	Zolbetuximab	Zolbetuximab monotherapy was well tolerated and exhibited antitumor activity in patients with CLDN18.2-positive advanced gastric and gastroesophageal junction adenocarcinoma.
FAST	NCT01630083	2b	Zolbetuximab plus epirubicin, oxaliplatin, and capecitabine (EOX) versus EOX alone	Significant survival benefits with zolbetuximab addition in HER2-negative metastatic gastric cancer.
SPOTLIGHT	NCT03504397	3	Zolbetuximab plus mFOLFOX6	Significantly increased median PFS and median OS in patients with CLDN18.2-positive and HER2-negative advanced gastric and gastroesophageal junction adenocarcinoma cancers.
GLOW	NCT03653507	3	Zolbetuximab plus CAPOX as a first-line treatment	Zolbetuximab addition significantly improved median PFS and median OS in patients with CLDN18.2-positive, HER2-negative, locally advanced unresectable, or metastatic gastric or gastroesophageal junction adenocarcinoma cancer.
Chimeric antigen receptor T-cells targeting claudin18.2 in solid tumors	NCT03874897	1	CLDN18.2-targeted CAR-T cells	CLDN18.2-targeted CAR-T cells showing promising efficacy with an acceptable safety profile in patients with heavily pretreated, CLDN18.2 positive gastric cancer.
VIKTORY	NCT02299648	NA	Sequencing of ctDNA	ctDNA can be used for identifying targetable alterations for personalized treatment in the context of metastatic GC, and ctDNA prediction of treatment response based on ctDNA is earlier than radiological assessments.
SHINE	NCT01457846	2	AZD4547	AZD4547 failed to improve median PFS compared to paclitaxel.
YO28252	NCT01590719	2	Onartuzumab plus mFOLFOX6 versus mFOLFOX6 alone	Onartuzumab failed to demonstrate improved clinical outcomes when combined with mFOLFOX6 in metastatic HER2-negative gastric or gastroesophageal junction cancer.
FIGHT	NCT03343301	2	Bemarituzumab plus mFOLFOX6	Bemarituzumab did not significantly improve median PFS in combination with mFOLFOX6, but a post hoc analysis revealed prolonged median OS, particularly in patients with FGFR2b overexpression.
FORTITUDE-102	NCT05111626	3	Bemarituzumab plus chemotherapy and nivolumab	Ongoing study—no results reported up to now.
RILOMET-1	NCT01697072	3	Rilotumumab plus epirubicin, cisplatin, and capecitabine	Studies were prematurely terminated due to an observed increase in mortality among participants receiving rilotumumab.
RILOMET-2	NCT02137343	3	Rilotumumab plus cisplatin and capecitabine	Studies were prematurely terminated due to an observed increase in mortality among participants receiving rilotumumab.
A phase 3 randomized, double-blinded, placebo-controlled study of ARQ 197 plus erlotinib	NCT01377376	2	ARQ 197 plus erlotinib	No significant clinical benefit in patients with metastatic gastric cancer.
A study of AMG 337 in subjects with advanced solid tumors	NCT01253707	1	AMG 337	No significant clinical benefit in patients with advanced solid tumors.
Study of GSK 1363089 in metastatic gastric cancer	NCT00725712	2	GSK1363089	No significant clinical benefit in patients with metastatic gastric cancer.
GOLD	NCT01924533	3	Olaparib plus paclitaxel	No significant survival advantage from the addition of olaparib to taxane in the second-line treatment of advanced gastric cancer.
HERIZON	NCT02795988	2	HER-Vaxx plus standard chemotherapy versus standard chemotherapy alone	HER-Vaxx is safe and provides relevant clinical benefit over standard chemotherapy in patients with HER2/neu overexpressing metastatic or advanced gastric and gastroesophageal junction cancer who were naïve to HER2 therapy.
